# Derotational Osteotomy and Plate Fixation of the Radius and Ulna for the Treatment of Congenital Proximal Radioulnar Synostosis

**DOI:** 10.3389/fsurg.2022.888916

**Published:** 2022-04-13

**Authors:** Yimurang Hamiti, Maimaiaili Yushan, Ainizier Yalikun, Cheng Lu, Aihemaitijiang Yusufu

**Affiliations:** Department of Microrepair and Reconstructive Surgery, The First Affiliated Hospital of Xinjiang Medical University, Urumqi, Xinjiang, China

**Keywords:** congenital, radioulnar synostosis, derotation, forearm deformity, osteotomy

## Abstract

**Purpose:**

To evaluate the clinical outcomes of derotational osteotomy followed by plate fixation at the radius and ulna for the treatment of congenital proximal radioulnar synostosis.

**Methods:**

A total of 10 eligible patients (12 forearms) with congenital proximal radioulnar synostosis were admitted to our institution from January 2013 to January 2016 and treated by radioulnar derotational osteotomy followed by plate fixation. There were 5 males and 5 females with an average age of 5.4 ± 2.0 (3–9) years old. The average forearm position was 56.67 ± 14.36° (range, 40°–80° pronation) in pronation before surgery. According to the classification system of Cleary and Omer, 3 forearms were categorized as type II, 7 as type III, and 2 as type IV. The pre- and postoperative forearm function was recorded and evaluated by the Failla scoring system.

**Results:**

All included patients were successfully followed up for an average time of 73.90 ± 8.24 months (range, 61–84 months). The mean achieved correction of the forearm was 53.33° ± 12.67° (range, 35°–70°). The average final position was 3.33° ± 14.98° (range, 20° of supination to 25° of pronation) in pronation. Bony union was achieved in a mean of 10.38 ± 1.25 weeks (range, 8.4–12.3 weeks) with no loss of correction. There were no incidences of nonunion, osteomyelitis, or neurologic or circulatory complications. The preoperative functional results were good in 1 forearm, fair in 8 forearms and poor in 3 forearms. In terms of final follow-up functional evaluations, 3 forearms were excellent, 6 forearms were good, and 3 forearms were fair.

**Conclusions:**

Congenital proximal radioulnar synostosis can be successfully treated using derotational osteotomy and plate fixation of the radius and ulna, which is an effective method with fewer postoperative complications and expected clinical outcomes.

## Introduction

Congenital radioulnar synostosis (CRUS) is one of the uncommon congenital upper limb deformities of the forearm that refers to an aberrant stiff connection between the proximal radius and ulna as a result of chromosomal disorders and other abnormalities of the upper limb (1). The majority of patients show bilateral involvement with the deformity (2). Generally, the forearm is fixed in the pronation position, which is the most noticeable characteristic of this deformity. With mild deformity, the ipsilateral shoulder and wrist joints can effectively compensate for any functional impairment (3). However, daily activities may be significantly restricted in cases with a severe permanent pronation deformity or whenever bilateral involvement is present (1, 2).

The treatment of CRUS is widely debated. Although It has been reported in the literature that surgery is essential to improve the quality of life of children with CRUS, there are no specific guidelines available to help the surgeon decide between nonsurgical and surgical treatment options for CRUS since each patient’s situation is different (2). As a result, surgical considerations should be based on functional impairment in addition to a significant pronation deformity.

Several surgical options, such as restoration of rotation and acute or staged derotation, have been described in the literature. Nevertheless, there is no common agreement on the surgical technique (1–27). A commonly accepted surgical management approach to treating CRUS is derotational osteotomy (3, 10–17, 19–26). This procedure moves the forearm from hyperpronation to a more functional position, which reduces pronation limits and makes it easier for patients to perform daily activities with the assistance of the shoulder and wrist joints. There are several types of osteotomy, including a single osteotomy of the radial diaphysis (23–25), a derotational osteotomy of the ulna and radius (10, 11, 19–21), and a synostosis-site derotational osteotomy (12, 22).

In this study, we present clinical outcomes in 10 patients (12 forearms) with CRUS who underwent operation using derotational osteotomy and plate fixation at the radius and ulna, and summarized our experiences.

## Materials and Methods

### Study Design

This was a single-center retrospective case series. All surgeries were carried out by members of the same surgical team. Medical records were analyzed by two surgeons (MY and AYu) for data, including radiological findings, operation records, and medical files. Demographic and baseline information such as age, gender, affected side and follow-up time was recorded by three surgeons (YH, AYa and CL). A total of 10 eligible patients (12 forearms) with CRUS who were treated by derotational osteotomy and plate fixation between January 2013 and January 2016 were included. Patients with the posttraumatic type of radioulnar synostosis were excluded. Patients who were lost to follow-up or not willing to participate in the present study were also excluded.

### Preoperative Assessment

All patients underwent preoperative radiographic examinations and were scored according to the Failla classification criteria (8). The classification system was based on 15 tasks described by Morrey et al. (28). If all tasks were completed, performance was graded as excellent; 10–14 points, good; 6–9 points, fair; and 3 points or less, poor. According to the classification system of Cleary and Omer, congenital radioulnar synostosis can be classified based on the radiographic appearance of the synostosis and radial head reduction (9). Type I synostosis is characterized by a decreased and normal-appearing radial head and does not involve bone. Types II, III, and IV have osseous synostosis. The radial head of type III is hypoplastic and posteriorly displaced. Type IV features a hypoplastic and anteriorly dislocated radial head. In our series, 3 forearms were categorized as type II, 7 as type III, and 2 as type IV.

### Surgical Technique

The patient was placed in a supine position under general anesthesia. A tourniquet was applied to the upper arm. A 5-centimeter longitudinal incision was made over the distal radius. The skin and subcutaneous superficial and deep fascia were gradually separated. The radius shaft was subperiosteally exposed, and a transverse osteotomy was conducted. Then, a 5-centimeter longitudinal incision was made in the proximal ulna distal to the synostosis site, and a transverse osteotomy was performed. Sutures were used to gently close the periosteum. The forearm was gradually rotated from the pronation position to the correction position, a range of 20 degrees supination to 20 degrees pronation. When performing osteotomy and rotating the forearm, care was taken to avoid nerve and vessel injury. Fixation of the osteotomy site was accomplished using a locking plate and screw system. The skin incision was subsequently closed. A cast was applied to fix the forearm in the correction position. Additionally, peripheral blood circulation and skin temperature were constantly monitored.

### Postoperative Management

Postoperatively, the forearm was elevated, and peripheral blood circulation and forearm edema were assessed for neurovascular complications. Regular dressing changes were needed to avoid the occurrence of infection in the operative area and around the wound. Monthly radiographic exams were performed for regular assessments. After 3–4 weeks, the plaster cast was removed. Upper extremity exercises were performed to improve daily functioning. The internal fixation was removed after the osteotomy sites were completely healed.

### Outcome Evaluation

Follow-up was conducted monthly at the outpatient clinic by a specially trained surgeon of our team as clinical visits to evaluate the incidence of complications. Postoperative X-rays were used to assess bone union at the osteotomy site. To assess the surgical outcome, the axial position of the forearm was measured pre- and postoperatively using a goniometer to detect improvements in forearm function. Preoperative and postoperative forearm functions were also examined using the categorization system devised by Failla et al. (8).

### Statistical Analysis

Statistical analysis was performed using SPSS 25.0 statistical software (IBM, USA). Continuous data were expressed as the mean ± standard deviation (SD). Differences between pre- and postoperative scores in the Failla classification were analyzed with the paired t-test and Wilcoxon matched-pair signed-rank tests. A *p*-value <0.05 was considered statistically significant.

## Results

**Figure 1:**
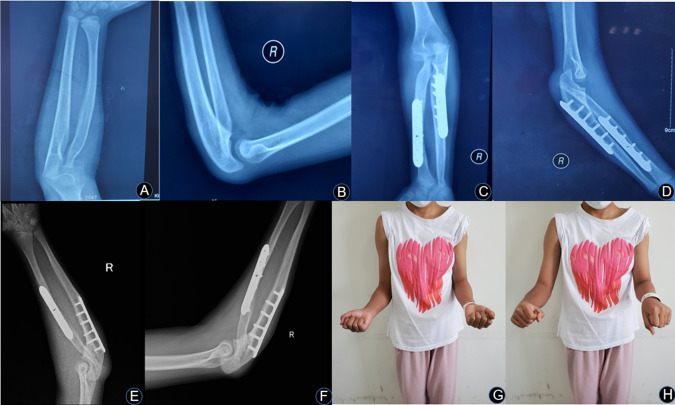
(**A,B**) A 7-year-old girl who had right proximal radioulnar synostosis that was fixed in the pronation position. Her preoperative Failla score was 8 points. Preoperative anteroposterior and lateral views of the right proximal radioulnar synostosis show a Cleary and Omer type III deformity. (**C,D**) Bone union was achieved at the osteotomy site after 3 months of surgery. (**E,F**) Excellent bone result is shown on anteroposterior and lateral views of X-ray at 7 years after the operation. (**G,H**) The rotational function of the right forearm was significantly improved at the last visit. The patient’s daily activities were greatly improved.

A total of 10 patients, 5 male and 5 female, with an average age of 5.4 ± 2.0 (3–9) years, were enrolled in this study. The details of the patients are shown in Table 1. The average time of follow-up was 73.90 ± 8.24 months (range, 61–84 months). The average forearm position was 56.67 ± 14.36° (range, 40°–80° pronation) in pronation before surgery. The average final position was 3.33° ± 14.98° (range, 20° of supination to 25° of pronation) in pronation. The mean achieved correction of the forearm was 53.33° ± 12.67° (range, 35°–70°) (*t* = 14.578, *p* < 0.01). The mean Failla categorization scores improved from 6.33 ± 3.28 points (range, 1–11 points) preoperatively to 11.92 ± 2.94 points (range, 7–15 points) postoperatively. The difference was statistically significant (*t* = −21.482, *p* < 0.01). The preoperative functional results were good in 1 forearm, fair in 8 forearms and poor in 3 forearms. In terms of final follow-up functional evaluations, 3 forearms were excellent, 6 forearms were good, and 3 forearms were fair (Table 2). All forearms had good radiological healing with an average time of 10.38 ± 1.25 weeks (range, 8.4–12.3 weeks). There were no incidences of nonunion, osteomyelitis, or neurologic or circulatory complications. A typical case is shown in Figure 1.

**Table 1:** Individual patient data.

Case	Gender	Age (years)	Affected Side	Forearm Pronation (degrees)		Correction of forearm rotation (degrees)	Failla Classification Criteria (score/classification)		Duration of Bone Union (weeks)	Follow-up Period (months)
				Preoperative	Postoperative		Preoperative	Postoperative		
1	M	3	Left	40	0	40	9/Fair	15/Excellent	9.9	75
			Right	60	20	40	6/Fair	11/Good	10.9	
2	F	7	Right	60	10	50	8/Fair	14/Good	12.1	84
3	M	3	Left	50	15	35	8/Fair	14/Good	10.5	61
			Right	40	−20	60	11/Good	15/Excellent	9.7	
4	M	5	Left	50	5	45	7/Fair	13/Good	8.5	77
5	M	9	Right	80	10	70	1/Poor	7/Fair	12.3	65
6	F	3	Right	40	−15	55	9/Fair	15/Excellent	9.7	73
7	F	5	Right	60	0	60	7/Fair	11/Good	8.4	82
8	M	7	Left	70	25	45	2/Poor	8/Fair	10.5	63
9	F	6	Left	50	−20	70	7/Fair	12/Good	11.7	78
10	F	6	Right	80	10	70	1/Poor	8/Fair	10.3	81

**Table 2:** Comparison of results of preoperative and postoperative data (mean ± SD).

	Preoperative	Postoperative	*P*-value
Failla Classification Criteria (points)	6.33 ± 3.29	11.92 ± 2.94	<0.01
Forearm Pronation (degrees)	56.67 ± 14.36	3.33 ± 14.98	<0.01

## Discussion

CRUS is an uncommon congenital forearm deformity considered to be caused by the failure of longitudinal segmentation and formation of the proximal radius and ulna during the seventh week of development (1, 2, 10–12). The clinical characteristics of CRUS are highly diverse and the majority of patients present bilateral, yet asymmetric, affection. The noticeable range of motion will be limited in severe persistent pronation deformities with pathologic fusion (1, 2).

The surgical indications for CRUS are controversial. Simmons et al. (13) advised that pronation greater than 60 degrees was an absolute indication for operative intervention. Ogino et al. (14) proposed that a fixed pronation of 60 degrees, based on functional limitation, constituted a relative surgical indication. However, Cleary et al. (9) believed that the majority of patients are quite functional and do not require surgical intervention. It is difficult to generalize the reported indications for CRUS surgery since each patient’s situation is different. As a result, surgical considerations should be based on functional impairments in addition to a predetermined pronation level. In our study, all patients had a mean deformity of 56.67 ± 14.36° (range, 40°–80° pronation) in pronation and a significant disability.

There are several surgical options described in the literature, but there is no generally accepted method (1, 3, 10–24). A recent systematic review concluded that surgery is critical for improving the quality of life of children with CRUS. Each form of surgery, however, is linked with complications (2). Reconstruction of the proximal radioulnar joint by resecting the fusion site could theoretically restore forearm rotation and function. However, operative mobilization requires the expertise of a microvascular surgeon and extensive surgical dissections, and the outcomes have been unsatisfactory. Synostosis recurrence is common, even with the use of soft-tissue, metal, or plexiglass implants as an interposition option (1, 2, 15–17). Kanaya et al. (18) described seven instances of bone excision of synostosis with free vascularized tissue interposition and all patients had no recurrence of fusion and acquired rotation in the forearm at short-term follow-up. Due to the morbidity and difficulty involved with the interposition of free vascularized tissue and the dependability of simpler methods, the separation approach has not achieved widespread adoption.

It has been shown in the literature that derotational osteotomy is the most frequent CRUS surgery since it advances the forearm into a more functional position(1, 2). There are several types of osteotomy, including a single osteotomy of the radial diaphysis (23–25), a derotational osteotomy of the ulna and radius (10, 11, 19–21), and a synostosis-site derotational osteotomy (12, 22). Pei et al. (12) reported proximal radioulnar derotational osteotomy at the fusion site followed by plate fixation for the treatment of 31 patients (36 forearms). The mean correction achieved was 70.86 ± 9.58 (50–90) degrees with a mean follow-up duration of 55.19 ± 27.10 (24–123) months. Simcock et al. (22) performed derotational osteotomy at the synostosis site followed by K-wire fixation in 26 patients (31 forearms). The mean correction achieved was 77 degrees (range, 40–95 degrees), resulting in a mean final position of 8 degrees of pronation (range, 0–30 degrees). Fujimoto et al. (23) described rotational osteotomy at the diaphysis of the radius in 3 patients (4 forearms). In all patients, bone union was accomplished and there was no loss of the forearm rotation correction. Horii et al. (24) performed 35 radial diaphysis osteotomies. The average forearm position was improved from 72 degrees pronation before surgery to neutral after surgery, with the exception of 2 forearms. Satake et al. (25) published 9 patients (12 forearms) with CRUS who underwent simple rotational osteotomy of the radius shaft. At the last follow-up, the average correction of the forearm rotation angle was 55° (range, 30°–90°), and the average actual forearm position was 4° supination (range, 20° pronation–30° supination). In the present study, all patients underwent derotational osteotomy of the proximal ulna and the distal radius followed by plate internal fixation, and the mean achieved correction of the forearm achieved was 53.33° ± 12.67° (range, 35°–70°).

The ideal time for surgery remains controversial. Several authors have suggested an age range of 3–6 years (21, 23). They reasoned that the osteotomy is straightforward at these ages and is likely to result in adequate remodeling of the radius and ulna. Horii et al. (24) recommended that the optimal age ranges from 4 to 9 years. The explanation for this is that the sturdy periosteum at these ages can support the cut radius and assist callus formation, and nerves and vessels can endure the torsional deformity, avoiding postoperative problems. In the present study, all patients received surgical treatment between the ages of 3 and 9 years. Throughout the course of CRUS patients development, the soft tissue contracture of the forearm gradually increases, and clinical symptoms and manifestations become more apparent. Consideration of surgical treatment when severe deformities have already manifested already increases the difficulty of forearm deformity correction, surgical risks, and postoperative complications; therefore, early surgery can largely avoid these risks and drawbacks and improve postoperative outcomes.

The optimal position after derotation remains a controversial issue. Green et al. (26) recommended that the optimal position in bilateral cases was 30–45 degrees pronation in the dominant forearm and 20–35 degrees supination in the nondominant forearm. Supination of 10–20 degrees was optimal in unilateral situations. Several clinicians have proposed forearm supination of 0–20 degrees in the nondominant forearm and pronation of 0–30 degrees in the dominant forearm (10, 14, 21, 23). Other authors have advocated a position of 30 degrees of supination to 20 degrees of pronation in both forearms (11, 12, 19, 20, 22). The optimal position is determined by the patient’s involved side, dominance, and cultural environment. People have begun to use computers and mobile phones considerably more often in the last decade as communication device use has increased. In addition, people from various cultures use a variety of types of tableware. In Asian countries, people typically eat with chopsticks. Taking these findings into consideration, all forearms were adjusted to a range of 20 degrees of supination to 20 degrees of pronation in the present study, since compensatory motions at the shoulder and wrist allow the forearm to be placed optimally.

Complications of derotational osteotomy have been reported. Excessive soft-tissue constriction may lead to circulatory compromise, compartment syndrome, or nerve palsy, all of which have been recorded as serious complications following osteotomy through synostosis sites (11, 13, 14, 16, 26). The authors of three papers reported the loss of correction in their case series since the fixation of the forearms was accomplished using K-wire or cast immobilization only (10, 20, 21). Shingade et al. (19) reported delayed bone union in 2 of 28 cases. When performing osteotomy at the proximal ulna, it is better to avoid the major nutrient artery deliberately. In the present study, we performed osteotomies of both the radius and ulna at different levels and fixed them with plate and cast immobilization, and no complications occurred as previously described.

The authors acknowledge that there are limitations to this study. First, this study was conducted retrospectively at a single center and was therefore susceptible to selection and indication biases. Furthermore, the Failla system has not been widely used to evaluate the treatment outcome of CRUS. Additional prospective studies that use validated and standardized patient-reported outcomes measures are required to overcome methodological shortcomings in the future. Second, there was just one cohort, consisting of ten patients, and there was no control group. While a randomized, multicenter controlled study would be ideal, it would raise ethical concerns.

## Conclusions

The findings of this study suggest that using derotational osteotomy and plate fixation of the radius and ulna to treat the congenital proximal radioulnar synostosis is effective. It might be a valuable addition to resolve deformity issues. Further clinical studies with a longer follow-up period are necessary.

## Data Availability

The raw data supporting the conclusions of this article will be made available by the authors, without undue reservation
